# Mandibular Canal Course and the Position of the Mental Foramen by Panoramic X-Ray in Chilean Individuals

**DOI:** 10.1155/2018/2709401

**Published:** 2018-06-07

**Authors:** Gloria Cartes, Ivonne Garay, Naira Figueiredo Deana, Pablo Navarro, Nilton Alves

**Affiliations:** ^1^Master Program in Sciences, Minor in Morphology, Faculty of Medicine, Universidad de La Frontera, Temuco, Chile; ^2^Dental School, Universidad de La Frontera, Temuco, Chile; ^3^Master Program in Dentistry, Dental School, Universidad de La Frontera, Temuco, Chile; ^4^Research Centre in Dental Sciences (CICO), Dental School, Universidad de La Frontera, Temuco, Chile; ^5^Research Centre in Applied Morphology (CIMA), Dental School, Universidad de La Frontera, Temuco, Chile; ^6^Center of Excellence in Morphological and Surgical Sciences (CEMyQ), Universidad de La Frontera, Temuco, Chile

## Abstract

The object of this study was to analyse the morphology and morphometry of the mandibular canal (MC) course and the mental foramen (MF) position in relation to the inferior teeth by panoramic X-ray (PAN). Vertical linear measurements were taken of each hemimandible to obtain the length of the distances analysed. We studied the MF position in relation to inferior premolar roots and the relation between the MC and mandibular teeth roots (MCR). The MF was usually located between the apices of the first and second premolars in younger individuals and immediately below the apex of the inferior second premolar in older individuals. The MC evinced proximity to the third molar, and this relation was not affected by sex or age group. The distances analysed in this study presented a marked difference between gender, with larger values in males than in females. The variations which may occur between individuals and different populations make it essential for dentists and surgeons to plan carefully before procedures involving this region.

## 1. Introduction

The mandible is an irregular, symmetrical bone which makes up the lower third of the face. It consists of a body and two rami which extend from the body's posterior ends [1]. The mandibular canal (MC) starts at the mandibular foramen and extends through the body of the mandible, towards the median plane. The inferior alveolar vasculonervous bundle passes through the mandibular foramen [1]. In panoramic X-ray images, the MC appears as a dark band of radiolucence flanked by two radio-opaque lines [2]. The mandibular canal gives rise to another small canal which opens in the mental foramen (MF). During surgery, the MC is used as a reference point [3]. The MC is of particular importance for dentists and oral specialists, as it carries the portion of the mandibular nerve responsible for inferior lip and teeth innervation [4, 5]. Special care is required in some procedures in this region because a lesion to the related blood vessels may cause excessive damage and lesion to vital structures. Lesions to the inferior alveolar nerve may cause pain and alterations in the patient's sensitivity [6]. Knowledge about the morphology and topography of the mandibular canal is important when carrying out interventions in the mandible, in order to preserve anatomical structures which pass through it. Not only is anatomical knowledge about the region a contributory factor to success in some procedures such as successful local anaesthesia in the inferior alveolar nerve terminal branches [7], but also it may be a determining factor in reducing paresthesia and haemorrhage occurrence, as well as lowering the risk of complications during surgical procedures such as osteotomy and mandibular implant positioning [8]. Previous studies have shown that MC course and MF position may vary between different populations [4, 7]. Few studies have been carried out in Chilean individuals in this respect, and exact data is needed for this population. The object of this study was to analyse the morphology and morphometry of MC course and position and MF position in relation to inferior teeth in adult Chileans by panoramic X-ray (PAN), considering age and sex.

## 2. Material and Methods

We carried out a descriptive, retrospective, cross-sectional study. We examined 442 panoramic X-rays taken between June 2014 and June 2016 as part of the diagnosis or treatment planning for patients who attended the Dental Teaching Clinic at the Faculty of Dentistry at the Universidad de La Frontera, Chile.

The sample included patient X-rays divided into four groups by age and gender: females between 18 and 34 years; females aged 35 or more; males between 18 and 34 years; and males aged 35 or more. Inclusion criteria are as follows: (1) patients of either sex aged 18 or over; (2) panoramic X-rays which clearly showed the reference points determined for measuring the distances. Exclusion criteria are as follows: (1) previously extracted premolars; (2) in-progress or completed orthodontic treatment; (3) fractures or surgery in the area which might affect X-ray interpretation; (4) pathology or congenital anomaly which might affect X-ray interpretation; (5) X-rays of patients with badly positioned teeth in the relation between the MF and the inferior teeth; (6) MF absent or not visible in the PAN.

PANs were taken by PAX-400C orthopantomograph, Vatech Co., Korea, Series 003-0848. The linear measurements were taken with AutoCAD 2010 software.

### 2.1. Linear Measurements

**Figure 1:**
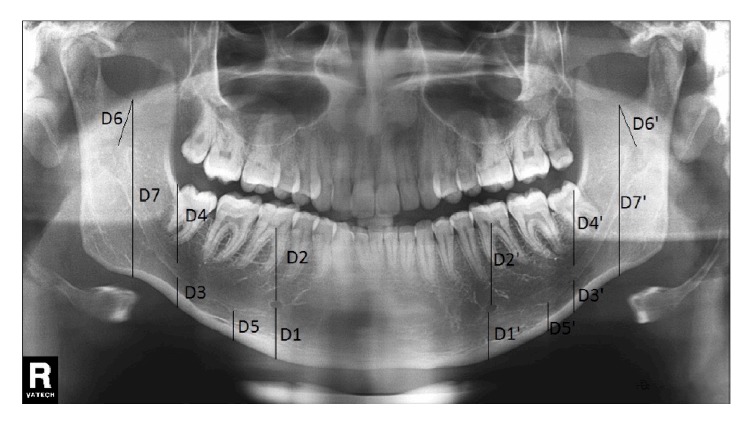
Panoramic X-rays illustrating the vertical linear measurements D1 to D7 on left and right sides (‘). D1: vertical distance from the inferior border of the mental foramen to the inferior limit of the base of mandible; D2: vertical distance from the superior border of the mental foramen to the superior limit of the highest alveolar ridge; D3: vertical distance from the inferior border of the mandibular canal to the inferior border of the base of mandible, on a vertical line through the mandibular ramus anterior border; D4: vertical distance from the superior border of the mandibular canal to the inferior limit of the oblique line, on a vertical line through the mandibular ramus anterior border; D5: vertical distance from the lowest point of the mandibular canal to the inferior limit of the base of mandible; D6: vertical distance from the lowest point of the mandibular notch to the mandibular foramen; D7: vertical distance from the lowest point of the mandibular notch to the inferior border of the mandibular ramus. The mean values were calculated for distances D1-D7, considering sex, side, and age group.

Vertical linear measurements were taken in digital PAN using specific points on the mandible as reference points. The following measurements were taken in each X-ray, following Amorim et al. [10] (Figure 1):

D1 is vertical distance from the inferior border of the mental foramen to the inferior limit of the base of mandible; D2 is vertical distance from the superior border of the mental foramen to the superior limit of the highest alveolar ridge; D3 is vertical distance from the inferior border of the mandibular canal to the inferior border of the base of mandible, on a vertical line through the anterior border of the mandibular ramus; D4 is vertical distance from the superior border of the mandibular canal to the inferior limit of the oblique line, on a vertical line through the anterior border of the mandibular ramus; D5 is vertical distance from the lowest point of the mandibular canal to the inferior limit of the base of mandible; D6 is vertical distance from the lowest point of the mandibular notch to the mandibular foramen; D7 is vertical distance from the lowest point of the mandibular notch to the inferior border of the mandibular ramus. The mean values were calculated for distances D1-D7, considering sex, side, and age group.

We also analysed the correlation between distances D1 to D7 based on four classifications: (a) measurements drawn approximately on a vertical (D1 x D2, D3 x D4, D6 x D7); (b) bilateral measurements D1 to D7 right x D1 to D7 left; (c) measurements taken above the mandibular canal (D2 x D4 x D6); (d) measurements taken below the mandibular canal (D1 x D3 x D5).

### 2.2. Relation between the Mandibular Canal and the Mandibular Teeth Roots (MCR)

**Figure 2:**
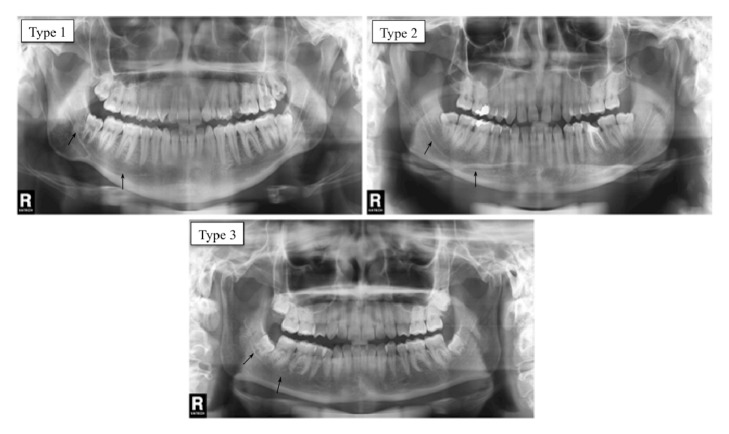
Panoramic X-rays illustrating relation types between mandibular canal and t inferior roots. Type 1: a relation of proximity exists between the mandibular canal and the third molar root. From this point the mandibular canal starts to diverge gradually from the roots. Type 2: no proximity between mandibular canal and roots. Type 3: proximity exists between mandibular canal and roots.

The MCR was classified into three types, following Madeira [17] (Figure 2): (1) a proximity relation exists between the mandibular canal and the third molar root. From this point the mandibular canal starts to diverge gradually from the roots; (2) no proximity exists between the mandibular canal and the roots; (3) a proximity relation exists between the mandibular canal and all roots. The percentage of each MCR type was calculated, and an analysis was carried out distinguishing sex, side, and age group.

### 2.3. Position of the Mental Foramen

**Figure 3:**
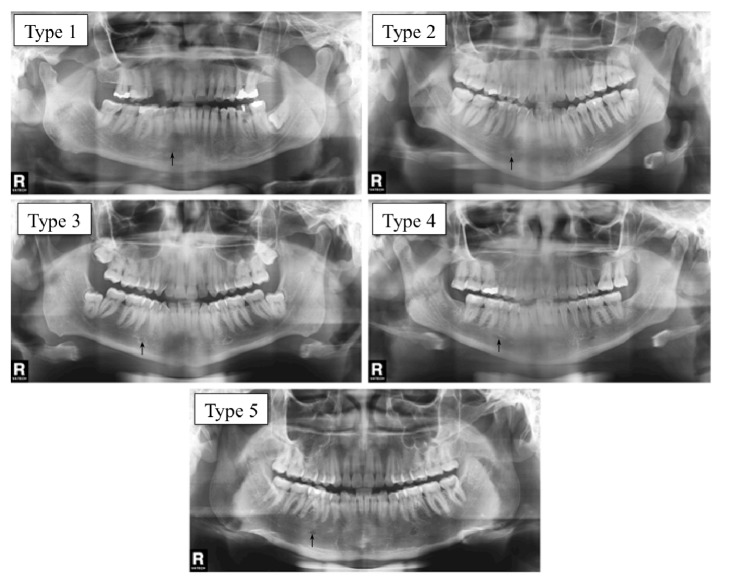
Panoramic X-rays illustrating the relation between the mental foramen (arrows) and inferior premolar roots. Type 1: anterior to the inferior first premolar. Type 2: below the inferior first premolar apex. Type 3: Between inferior premolar roots. Type 4: immediately below the inferior second premolar apex. Type 5: posterior to the inferior second premolar.

The relation between the mental foramen and inferior premolar roots was classified in five positions, following Madeira [17] (Figure 3): Type 1: anterior to the inferior first premolar; Type 2: below the inferior first premolar apex; Type 3: between the inferior premolar roots; Type 4: immediately below the inferior second premolar apex; Type 5: posterior to the inferior second premolar. The percentage of each MF position type was calculated, and an analysis was carried out distinguishing sex, side, and age group.

### 2.4. Statistical Analysis

The data were analysed by descriptive statistics (mean±SD). The Shapiro-Wilk test and Levene's test were applied for variance homogeneity. The statistical difference between mean values was obtained using Student's t-test for equality of means. All means were correlated using Pearson's correlation test and the results were classified with Pearson's rating scale. The Chi-squared test was used for qualitative variables. Statistical analysis was carried out using SPSS/PC + software, version 23.0, SPSS, Chicago, IL. The significance threshold was set at *α*=5%.

## 3. Results

Of the 442 panoramic X-rays examined, 262 were from females and 180 from males. Of the 262 females, 191 were aged between 18 and 34 years, mean 23.01 (±4.21 years); 71 were aged over 35 years, mean 44.52 (±6.93 years); of the 180 males, 145 were aged between 18 and 34 years, mean 23.08 (±4.29 years); and 35 were aged over 35 years, mean 47.29 (±9.95 years).

### 3.1. Linear Measurements D1-D7

The mean values for D1-D7 are given in Table 1, distributed by sex, side, and age group.

#### 3.1.1. Analysis between Age Groups

When distances D1 to D7 in females were compared between age groups and left/right sides, no statistically significant differences were found, except for D2, where the mean value was observed to be greater in females aged 18-35 years.

In males, distance D3 on both sides and distance D7 on the left side presented significantly greater values in individuals aged 18-34 years.

#### 3.1.2. Analysis by Sex

Statistical differences were found between males and females aged 18-34 years for all the distances, with males presenting greater mean values than females on both sides.

In the 35-and-over age group, statistical differences were found between males and females for the distances D1, D2, D6, and D7 on both sides and D5 on the right side, with males presenting greater mean values than females.

When the complete sample was analysed, a significant difference between males and females was observed for all distances (*p* ≤0.01), with males presenting greater mean values than females.

#### 3.1.3. Correlation between Distances

A moderate positive correlation was found between distances D6xD7, D1xD5, and D3xD5. D1, D4, and D6 presented moderate positive correlation between left and right sides, while D2, D3, D5, and D7 presented a high positive correlation between left and right sides. The correlations between the distances are given in Table 2.

### 3.2. Relation between the Mandibular Canal and the Roots of the Inferior Teeth (MCR)

There was no statistically significant difference in the MCR between sides. In both males and females the mandibular canal was most commonly close to the third molar. In males the second most common relation was no proximity between the mandibular canal and roots, while for females the second most common relation was proximity between the mandibular canal and all roots, with statistically significant differences (*p* ≤0.01). The percentages for females and males aged 18-34 years are presented in Table 3; no data are shown for males ≥35 years because the sample was not significant.

### 3.3. MF Position

Table 4 shows the percentages found for each MF type by sex, side and age group. A significant difference was found in MF position percentages by side among males aged ≥35 years, with Type 3 more frequent on the left side and Type 4 on the right. No statistical differences were found between sexes for the position of the MF.

## 4. Discussion

PAN is a quick, simple, and low-cost imaging technique requiring a low radiation dose. It is widely used in dentistry for diagnosis and presurgery planning [18–20]. X-ray interpretation is sometimes complicated due to the superimposition of anatomical structures [21], since the image is two-dimensional and subject to a degree of inaccuracy [18–20]. According to Kim et al. [22], PAN presents ±10% accuracy for linear measurements and morphological assessments. Muinelo-Lorenzo et al. [23] state that only 83.87% of the MF found by Cone-Beam computerised tomography are also observed in PAN; however other studies say that 100% of MF can be observed in PAN [24–26].

### 4.1. Analysis of Distances D1-D7

In this research, the distance from the MF to the base of mandible (D1) was shorter than the distance from the MF to the alveolar ridge, showing that the MF is closer to the base of mandible; this corroborates earlier studies [10, 12, 13, 27]. Furthermore, in females aged 35 or over and in males aged between 18 and 34 years, a negative correlation was observed between distances, corroborating the above finding. Alves [28] carried out a study in macerated mandibles of individuals with different degrees of edentulism and observed that the MF presented closest to the alveolar ridge in edentate mandibles, followed by mandibles with posterior edentation, and finally dentate mandibles; this shows that alveolar resorption can lead to a change in MF location in the mandible, bringing it closer to the alveolar ridge, leading in turn to a higher risk of complications for procedures in this region. In the present study, we found symmetry and marked difference between genders for these two distances, which are greater in males than in females, which corroborates previous studies [9, 11, 12, 14, 15]. Due to the marked gender difference, distances D1 and D2 are powerful indicators which can be used to determine the sex of individuals [12]. Age had no major impact on distances D1 and D2; an age group effect was only found for distance D2 on the right side, which was greater in younger females. The D1 value in our study of Chilean individuals was similar to that reported for Brazilian [10, 16] and Indian [11, 12, 15] individuals, greater than that reported for Iraqis [13, 14], and less than another study in Brazilians [9] (Table 5). Distance D2 was greater in Brazilian [10, 16] and Indian [12] individuals and similar in Iraqis [13] when compared to the present sample of Chilean individuals (Table 5). It bears noting that knowledge of MF location in relation to the inferior border of the mandible, the alveolar ridge, and adjacent teeth is important for avoiding lesions to the mental nerve during procedures in the region, such as genioplasty, apical curettage in the inferior premolar area, surgical extractions, dental implants, mandibular fracture fixation, and periodontal surgery [29]. Furthermore, correct MF location helps in precise nervous blocking, avoiding complications due to nerve lesions [29].

Males presented larger mean values for distances D3, D4, and D5, with significant gender differences. D3 was slightly larger in younger males than in those aged 35 or over, in contrast to findings among females; other authors have reported higher values in males [13]; however they did not carry out analysis by age group. In the present study, distance D3 was fairly similar to measurements found by other authors in Brazilian and Iraqi individuals [10, 13]. The mean value for D5 in our sample was similar to that found by de Paula et al. [16] in Brazilians and by Jayam et al. [15] in Indians and greater than in Amorim et al. [10] in Brazilians (Table 5). In the present study, distances D3 and D5 presented a moderate to high positive correlation, suggesting that mandibular canal curvature increases or decreases proportionally in those two regions. D4 in our study was higher than the values found in Brazilian [10] and Iraqi individuals [13]. We observed that both D3 and D4 presented important gender differences in younger individuals, with greater values in males than in females; this finding disagrees with earlier studies [10, 13]. The low to moderate negative correlation between these two distances suggests a variation in MC course, since when one of the distances increases, the other decreases; when D4 decreases and D3 increases, MC is positioned closer to the inferior third molar root.

The mandibular foramen is the opening through which the inferior alveolar vasculonervous bundle enters the mandibular canal [1]. The mandibular foramen position is used as a parameter for safe interventions involving the mandibular ramus region, including surgery and anaesthesia [30], and this helps reduce complications due to inferior alveolar vasculonervous bundle lesions [31]. In a study of macerated mandibles in Brazilian individuals, Alves and Deana [32] performed morphological analysis of the mandibular lingula superoinferior position, an anatomical structure which forms the medial limit of the mandibular foramen [1]. They reported that the lingula was located in the superior or middle third of the ramus, 1 mm away from the intersection line of these two-thirds. Fontoura et al. [33] carried out a study of macerated mandibles and X-rays to determine the mandibular foramen location and concluded that it is located almost completely in the middle third of the mandibular ramus. According to Trost et al. [34] the posterior and superior thirds of the mandibular ramus constitute a “safety zone” where the mandibular foramen is unlikely to be located. However some anatomical variations, such as the accessory mandibular foramen [35] or superior location of the mandibular foramen being very close to the mandibular notch [36], may increase inferior alveolar nerve (IAN) complication risks [35]; it is therefore essential to plan properly before carrying out interventions in the region, and panoramic X-ray can be a useful tool for correctly locating important structures such as the mandibular foramen. In our study, distance D6, the vertical distance from the lowest point of the mandibular notch to the mandibular foramen, was shorter than that reported by Amorim et al. [10] and de Paula et al. [16] in their studies in Brazil, than Ghouse et al. [12] in India and Rashid and Ali [13] in Iraq. Distance D6 was significantly greater in males than in females [10, 12, 13, 16] and is considered an important measurement for determining sex [12]. It should be noted that the mean values found for D6 in Chileans were significantly smaller than those reported for Brazilian [10] and Iraqi individuals [13, 14] and closer to those reported for Indians [12] (Table 5). In the present study, we found no age-related differences, showing that the mandibular foramen does not change its position vertically with age and corroborating other studies [12, 13, 37]. However, we note that previous studies in macerated mandibles did find an association between age and mandibular foramen location, with a more superior location found in younger males compared to older males and a more posterior location found in younger females compared to older females [35]. Sex, age, and ethnic group must be considered in surgical interventions or anaesthetic blocking, in order to reduce complication risks in this region [32, 35].

Based on our study we can state that distance D7 presented a marked gender difference, since values found for males were expressively greater than for females, corroborating previous studies [10, 12, 14]; this is an important measurement for determining sex [12, 14]. The mean values found for D7 in our study were smaller than those found for Brazilians [10], Indians [12], and Iraqis [13] (Table 5). In males we found differences between age groups, with greater values among younger individuals than in older groups, disagreeing with other studies [10]. A moderate positive correlation was found between D6 and D7 in all groups and sides, except on the left side in older males and females. The correlation between these two measurements suggests that distances from mandibular foramen to the mandibular notch and mandibular ramus height tend to increase or decrease proportionally, while the mandibular foramen remains in the same general position.

### 4.2. Relation between Mandibular Canal and Roots (MCR)

Variations in mandibular canal course are very frequent [2], and locating them is necessary to avoid lesions to the inferior alveolar nerve in procedures such as dental implants, dental anaesthesia, and mandibular osteotomy [38]. The MC is generally in contact with the inferior third molar alveolus base, and its distance from root apices of the other teeth increases gradually, as corroborated by the present work and previous studies [1, 10, 14]. The inferior alveolar vasculonervous bundle passes through the MC [1], and IAN proximity to the inferior third molar root is associated with neurosensorial complications after surgery to extract the inferior third molar [39]. According to Valmaseda-Castellón et al. [40], the radiologic relationship between third molar roots and the mandibular canal increases the risk of damage to the IAN, which may have a marked impact on quality of life [41], through paresthesia or anaesthesia in the inferior lip, chin, and buccal gingivae [40]. The risk of IAN deficit is increased in inferior third molar surgery when its impaction is horizontal, as compared to other impaction types [42]. Furthermore, impaction degree is also a factor which aggravates the risk of nervous lesions [42, 43]. In the present study, we observed that MCR was not affected by gender, agreeing with studies by Amorim et al. [10], Rashid and Ali [14], and Falkine et al. [44] and corroborating results in Cheung et al. [39], who found no association between sex and increased risk of IAN lesion in third molar surgery. Furthermore, in our study we found no association between age and MCR, corroborating previous studies [10]; however it must be noted that the literature is contradictory with respect to increased risk associated with age, since some studies have reported that age is a determinant for increased risk of IAN lesion [45] and patient morbidity during surgery [46], while other authors report no such findings [39, 40].

No-proximity relation between the mandibular canal and roots (Type 2) presented a lower percentage than Type 1, but higher than Type 3, with 12% on the right side and 12.9% on the left, which agrees with findings by Amorim et al. [10] for individuals aged up to 40.

Proximity relation between the mandibular canal and all roots (Type 3) was the least frequent in our study, with 11.2% on the right side and 12.5% on the left. Falkine et al. [44] found that this mandibular canal type was the second most frequent in their study population.

### 4.3. MF Position

MF is a very frequent anatomical structure, of great clinical importance. Knowledge of its anatomy is essential for avoiding complications during clinical and surgical procedures in this region [47]. In a literature review, Ceballos et al. [47] reported that MF was present in 95.2% of PANs analysed, being observed more frequently on the left side than the right. Furthermore, these authors stated that the MF is located between inferior premolar apices in 42.22% of cases, coincides with inferior second premolar roots in 33.98%, and is distal to the inferior second premolar root in 10.98% of PANs [47]. In a study performed with Chilean individuals, Fuentes et al. [48] found that MF was located on the longitudinal axis of the second premolar on the right side and between longitudinal axes of the first and second premolars on the left side; in our study, also in Chilean individuals, we found differences between sides only for males aged 35 or over, in which the MF was located between premolar roots on the right side and coincident with the second premolar on the left. In young individuals (18-30 years) in the United Kingdom, the most common MF position was between the first and second premolar apices [24], similar to our study for individuals of both sexes aged 18 to 34. The same position was found to be most common in other populations: Iraqi [7], Asiatic [25], Korean [49], and Indian [50]. In our study, the most common location in individuals aged 35 or over was immediately below the inferior second premolar apex, coinciding with other populations: Moroccan [51], Spanish [23], and Indian [50, 52]. No differences between sexes were found in MF position, corroborating previous studies [24, 26, 53]. The other three positions recurred less frequently; in the present study, Type 1 frequency ranged from 5.7% to 0%, Type 2 from 11.4 to 0%, and Type 5 from 17.1% to 3.7%. Amorim et al. [37] reported similar results, with values generally below 10%, except for Type 5 in F2, which reached 13.16%. Considerably lower percentages were reported by Almeida et al. [9], who found that Types 1 and 2 represented only 5% and 1% of cases, respectively, while no case of Type 5 was found.

## 5. Conclusions

The MF is generally located between the first and second premolar apices in younger individuals and immediately below the inferior second premolar apex in older individuals. The MC presented proximity to the third molar, and this relation was not affected by sex or age group. Distances analysed in this study presented symmetry and marked gender differences, with larger values found in males than in females. However, age did not seem to exert a strong influence on these measurements. We found a significant difference compared to other populations, suggesting that MC course varies in different ethnic groups. Variations which may occur between individuals and different populations make it essential for dentists and surgeons to plan carefully before procedures in this region.

## Figures and Tables

**Table 1:** Mean value (in millimetres) and standard deviation (SD) of distances D1 to D7 on the right and left sides for males and females in age groups 18-34 years and ≥35 years.

Distances	Females (18-34 years)		Females (≥35 years)		Males (18-34 years)		Males (≥35 years)	
	Average	SD	Average	SD	Average	SD	Average	SD
D1^R^	11.33^†^	±1.43	11.56^†^	±1.32	12.85^†^	±1.86	12.82^†^	±1.34
D1^L^	11.37^†^	±1.54	11.63^†^	±1.67	13.10^†^	±1.95	13.02^†^	±1.32
D2 ^R^	15.83^a†^	±2.1	14.91^a†^	±1.96	17.19^†^	±2.63	16.56^†^	±2.46
D2 ^L^	15.76^†^	±2.25	15.19^†^	±2.15	16.80^†^	±2.32	16.39^†^	±1.98
D3 ^R^	8.11^†^	±2.22	7.84	±1.82	9.03^†a^	±2.37	8.04 ^a^	±2.33
D3 ^L^	7.78^†^	±2.04	7.55	±2.16	8.60^†a^	±2.16	7.77 ^a^	±1.98
D4 ^R^	16.03^†^	±2.95	15.52	±2.87	17.24^†^	±3.34	16.55	±3.15
D4 ^L^	16.12^†^	±2.84	15.77	±3.1	17.13^†^	±3.00	16.84	±3.29
D5 ^R^	7.71^†^	±1.84	7.37^†^	±1.76	8.47^†^	±2.05	8.19^†^	±1.85
D5 ^L^	7.60^†^	±1.84	7.26	±2.02	8.54^†^	±2.32	7.86	±1.65
D6 ^R^	14.13^†^	±2.34	14.03^†^	±2.23	15.9^†^	±2.67	15.73^†^	±2.77
D6 ^L^	14.07^†^	±2.35	14.29^†^	±2.14	15.99^†^	±2.91	15.91^†^	±2.42
D7^R^	44.65^†^	±3.16	43.78^†^	±3.56	49.21^†^	±4.11	47.75^†^	±3.76
D7 ^L^	44.63^†^	±3.28	43.87^†^	±3.15	49.44^†a^	±3.87	47.93^†a^	±3.69

^a^Statistically significant difference between age groups. ^†^Statistically significant difference between sexes. ^R^Right. ^L^Left

**Table 2:** Correlation between distances, by sex and age group. Separate information by side is presented where there are significant differences.

Sex/age	Females 18-34 years	Females ≥35 years	Males 18-34 years	Males ≥35 years
Distances measured below the mandibular canal				
D1 x D3	0.29^*∗*^	0.01	0.32^*∗*^	0.12
D1 x D5	0.39^*∗*^	0.33^*∗*^	0.41^*∗*^	0.34
D3 x D5	0.71^*∗*^	0.46^*∗*^	0.52^*∗*^	0.61^*∗*^

Distances measured above the mandibular canal				
D2 x D4	0.10^*∗*^	0.19	0.10	0.23
D2 x D6	0.08	0.13	0.03	0.06
D4 x D6	0.20^*∗*^	0.18	0.21	0.16

Distances measured along the same vertical line				
D1^R^ x D2^R^	-0.07	-0.02	-0.29^*∗*^	0.06^*∗*^
D1^L^ x D2^L^		-0.32^*∗*^		-0.01^*∗*^
D3^R^ x D4^R^	-0.38^*∗*^	-0.50^*∗*^	-0.38^*∗*^	-0.25^*∗*^
D3^L^ x D4^L^				-0.42^*∗*^
D6^R^ x D7^R^	0.51^*∗*^	0.58^*∗*^	0.54^*∗*^	0.68^*∗*^
D6^L^ x D7^L^		0.32^*∗*^	0.64^*∗*^	0.33^*∗*^

^*∗*^Statistically significant.

**Table 3:** Percentage for each mandibular canal relation type with inferior roots, considering sex, side, and age group.

	Females (18-34 years)		Females (≥35 years)		Males (18-34 years)	
	Right	Left	Right	Left	Right	Left
Type 1	72.9%	72.9%	68.8%	62.5%	80.9%	75%
Type 2	7.3%	5.2%	18.8%	18.8%	12.8%	12%
Type 3	19.8%	21.9%	12.5%	18.8%	6.4%	7%

**Table 4:** Percentage of each mental foramen position type on the right and left sides for males and females in age groups 18-34 years and ≥35 years.

Sex, side and age group	Mental foramen Position				
	Type 1	Type 2	Type 3	Type 4	Type 5
Females^R^ (18-34 years)	2.1%	3.2%	54.0%	35.3%	5.3%
Females^L^ (18-34 years)	4.3%	1.6%	54.0%	36.4%	3.7%
Females^R^ (≥35 years)	0.0%	2.9%	35.3%	45.7%	17.1%
Females^L^ (≥35 years)	1.4%	2.9%	32.9%	45.7%	17.1%
Males^R^ (18-34 years)	0.7%	4.3%	50.4%	36.2%	8.5%
Males^L^ (18-34 years)	3.5%	3.5%	48.9%	39%	5.0%
Males^R^ (≥35 years)	5.7%	11.4%	20.0%	54.3%	8.6%
Males^L^ (≥35 years)	2.9%	0.0%	42.9%	45.7%	8.6%

^R^Right side. ^L^Left side.

**Table 5:** Mean values (mm) for distances D1-D7 reported in the literature and in the present study.

Author	Sample		Origin	Age	Side	D1		D2		D3		D4		D5		D6		D7	
	M	F				M	F	M	F	M	F	M	F	M	F	M	F	M	F
Almeida Filho et al. [9]	35	65	Brazil	≥18	R	17.80	16.38	-	-	-	-	-	-	-	-	-	-	-	-
					L	17.51	16.18	-	-	-	-	-	-	-	-	-	-	-	-

Amorim et al. [10]	101	199	Brazil	18-40	-	11.81	10.75	18.54	17.14	8.93	8.49	14.71	14.08	6.94	6.86	24.92	23.10	50.12	46.91
				≥40	-	11.91	10.62	17.44	16.53	8.80	8.74	-	12.45	7.32	6.87	25.07	23.74	48.52	45.41

Chandra et al. [11]	100		India	18-62	R	12.67	11.46	-	-	-	-	-	-	-	-	-	-	-	-
					L	12.58	11.25	-	-	-	-	-	-	-	-	-	-	-	-

Ghouse et al. [12]	60		India	20-49	R	13.73		18.79		-	-	-	-	-	-	15.72		56.58	
					L	13.79		18.07		-	-	-	-	-	-	16.27		56.68	
	20	20		20-29	-	14.47	13.60	18.57	18.31	-	-	-	-	-	-	19.22	19.75	55.08	51.63
	20	20		30-39	-	14.94	12.60	18.40	18.85	-	-	-	-	-	-	16.09	15.12	65.38	60.98
	20	20		40-49		14.56	12.38	19.48	16.97	-	-	-	-	-	-	13.65	12.14	56.66	50.36

Rashid and Ali [13, 14]	50	50	Irak	20-29	-	9.63	8.81	17.80	15.95	-	-	-	-	-	-	25.53	23.36	50.90	46.52
	50	50		30-39	-	10.60	9.84	17.88	16.82	-	-	-	-	-	-	24.92	23.19	50.31	46.17
	50	50		40-49	-	9.96	9.07	16.65	15.27	-	-	-	-	-	-	24.81	23.08	49.91	45.86
	300			-	R	9.63		16.87		9.34		15.27		-	-	24.15		48.28	
	300			-	L	9.68		16.89		9.3		15.33		-	-	24.16		48.29	

Jayam et al. [15]	25	25	India	20-30	-	13.04	12.62	-	-	-	-	-	-	7.50	7.28	-	-	-	-
	25	25		30-40	-	13.93	14.51	-	-	-	-	-	-	7.84	7.70	-	-	-	-

Simpson et al. [16]	21		Brazil	≥18	R	13.42		19.10		-	-	-	-	8.42		18.96		-	-
					L	13.42		18.89		-	-	-	-	7.78		18.46		-	-

Present study	191	1ç	Chile	18-34	R	12.85	11.33	17.19	15.83	9.03	8.11	17.24	16.03	8.47	7.71	15.9	14.13	49.21	44.65
		45																	
					L	13.10	11.37	16.80	15.76	8.60	7.78	17.13	16.12	8.54	7.60	15.99	14.07	49.44	44.63
	71	35		≥35	R	12.82	11.56	16.56	14.91	8.04	7.84	16.55	15.52	8.19	7.37	15.73	14.03	47.75	43.78
					L	13.02	11.63	16.39	15.19	7.77	7.55	16.84	15.77	7.86	7.26	15.91	14.29	47.93	43.87

M: males, F: females; R: right; L: left.

## Data Availability

The data used to support the findings of this study are available from the corresponding author upon request.

## References

[1] Alves N., Cândido P. (2016). *Anatomia para o curso de odontologia geral e específica*.

[2] Nortjé C. J., Farman A. G., Grotepass F. W. (1977). Variations in the normal anatomy of the inferior dental (mandibular) canal: a retrospective study of panoramic radiographs from 3612 routine dental patients. *The British Journal of Oral Surgery*.

[3] Lindh C., Petersson A., Klinge B. (1995). Measurements of distances related to the mandibular canal in radiographs. *Clinical Oral Implants Research*.

[4] Juodzbalys G., Wang H. L., Sabalys G. (2010). Anatomy of mandibular vital structures. Part I: mandibular canal and inferior alveolar neurovascular bundle in relation with dental implantology. *Journal of Oral & Maxillofacial Research*.

[5] Farman A., Nortjé C. J., Springer (2007). Panoramic radiographic appearance of the mandibular canal in health and in disease. *Panoramic Radiology*.

[6] Kim I.-S., Kim S.-G., Kim Y.-K., Kim J.-D. (2006). Position of the mental foramen in a Korean population: A clinical and radiographic study. *Implant Dentistry*.

[7] Al-Shayyab M. H., Alsoleihat F., Dar-Odeh N. S., Ryalat S., Baqain Z. H. (2015). The mental foramen i: Radiographic study of the anterior- posterior position and shape in iraqi population. *International Journal of Morphology*.

[8] Phillips J. L., Weller R. N., Kulild J. C. (1990). The mental forman: Part I. Size, orientation, and positional relationship to the mandibular second premolar. *Journal of Endodontics*.

[9] de Almeida Filho L. R., Reis H. S. M., Amadei S. U., Scherma A. P., Sousa D. M. (2011). Evaluation of the position of mental foramen in relation to the teeth and mandibule base in the conventional panoramic radiograph. *Brazilian Journal of Periodontology*.

[10] Amorim M. M., Borini C. B., Lopes S. L. P. D. C., Haiter-Neto F., Caria P. H. F. (2009). Morphological description of mandibular canal in panoramic radiographs of Brazilian subjects: Association between anatomic characteristic and clinical procedures. *International Journal of Morphology*.

[11] Chandra A., Singh A., Badni M., Jaiswal R., Agnihotri A. (2013). Determination of sex by radiographic analysis of mental foramen in North Indian population. *Journal of Forensic Dental Sciences*.

[12] Ghouse N., Nagaraj T., James L., Swamy N. N., Jagdish C. D., Bhavana T. (2016). Digital analysis of linear measurements related to the mental and mandibular foramina in sex determination. *Journal of Medicine, Radiology, Pathology and Surgery*.

[13] Rashid S., Ali J. (2011). Morphometric analysis of mandibular canal course and position in relation to gender and age of Iraqi sample using digital panoramic imaging. *Journal of Baghdad College Desntistry*.

[14] Rashid S. A., Ali J. (2011). Sex determination using linear measurements related to the mental and mandibular foramina vertical positions on digital panoramic images. *Journal Baghdad College Dentistry*.

[15] Jayam R., Annigeri R., Rao B., Gadiputi S., Gadiputi D. (2015). Panoramic study of mandibular basal bone height. *Journal of Orofacial Sciences*.

[16] de Paula J. S., Resende C. C., Domingos A. C., Devito K. L. (2011). Evaluación de la simetria bilateral del canal mandibular en las radiografías panorámicas. *Acta Odontológica Venezolana*.

[17] Madeira M. (2003). *Anatomia da Face: Bases Anátomo-Funcionais para a Prática Odontológica*.

[18] Garg A. K. (2007). Dental implant imaging: TeraRecons dental 3D Cone Beam Computed Tomography system. *Dental Implantology Update*.

[19] Garcia Silva M. A., Wolf U., Heinicke F., Gründler K., Visser H., Hirsch E. (2008). Effective dosages for recording Veraviewepocs dental panoramic images: analog film, digital, and panoramic scout for CBCT. *Oral Surgery, Oral Medicine, Oral Pathology, Oral Radiology, and Endodontology*.

[20] Vazquez L., Saulacic N., Belser U., Bernard J.-P. (2007). Efficacy of panoramic radiographs in the preoperative planning of posterior mandibular implants: a prospective clinical study of 1527 consecutively treated patients. *Clinical Oral Implants Research*.

[21] Liu T., Xia B., Gu Z. (2009). Inferior alveolar canal course: A radiographic study. *Clinical Oral Implants Research*.

[22] Kim H.-J., Lee H.-Y., Chung I.-H., Cha I.-H., Yi C.-K. (1997). Mandibular anatomy related to sagittal split ramus osteotomy in Koreans. *Yonsei Medical Journal*.

[23] Muinelo-Lorenzo J., Suárez-Quintanilla J.-A., Fernández-Alonso A., Varela-Mallou J., Suárez-Cunqueiro M.-M. (2015). Anatomical characteristics and visibility of mental foramen and accessory mental foramen: Panoramic radiography vs. cone beam CT. *Medicina Oral Patología Oral y Cirugía Bucal*.

[24] Currie C. C., Meechan J. G., Whitworth J. M., Carr A., Corbett I. P. (2015). Determination of the mental foramen position in dental radiographs in 18-30 year olds. *Dentomaxillofacial Radiology*.

[25] Gada S. K., Nagda S. J. (2014). Assessment of position and bilateral symmetry of occurrence of mental foramen in dentate Asian population. *Journal of Clinical and Diagnostic Research*.

[26] Gupta V., Pitti P., Sholapurkar A. (2015). Panoramic radiographic study of mental foramen in selected dravidians of south Indian population: A hospital based study. *Journal of Clinical and Experimental Dentistry*.

[27] Teerijoki-Oksa T., Jääskeläinen S. K., Forssell K. (2002). Risk factors of nerve injury during mandibular sagittal split osteotomy. *International Journal of Oral and Maxillofacial Surgery*.

[28] Alves N. (2009). Estudio de la localización del foramen mentoniano en mandíbulas maceradas con diferentes grados de edentulismo. *International Journal of Odontostomatology*.

[29] Al-Mahalawy H., Al-Aithan H., Al-Kari B., Al-Jandan B., Shujaat S. (2017). Determination of the position of mental foramen and frequency of anterior loop in Saudi population. A retrospective CBCT study. *The Saudi Dental Journal*.

[30] Yoshida T., Nagamine T., Kobayashi T. (1989). Impairment of the inferior alveolar nerve after sagittal split osteotomy. *Journal of Cranio-Maxillo-Facial Surgery*.

[31] Prado F. B., Groppo F. C., Volpato M. C., Caria P. H. F. (2010). Morphological changes in the position of the mandibular foramen in dentate and edentate Brazilian subjects. *Clinical Anatomy*.

[32] Alves N., Deana N. F. (2015). Morphological study of the lingula in adult human mandibles of brazilians individuals and clinical implications. *BioMed Research International*.

[33] Da Fontoura R. A., Vasconcellos H. A., Campos A. E. S. (2002). Morphologic basis for the intraoral vertical ramus osteotomy: Anatomic and radiographic localization of the mandibular foramen. *Journal of Oral and Maxillofacial Surgery*.

[34] Trost O., Salignon V., Cheynel N., Malka G., Trouilloud P. (2010). A simple method to locate mandibular foramen: Preliminary radiological study. *Surgical and Radiologic Anatomy*.

[35] Alves N., Deana N. F. (2014). Morphometric study of mandibular foramen in macerated skulls to contribute to the development of sagittal split ramus osteotomy (SSRO) technique. *Surgical and Radiologic Anatomy*.

[36] Cvetko E. (2014). Bilateral anomalous high position of the mandibular foramen: a case report. *Surgical and Radiologic Anatomy*.

[37] Amorim M. M., Prado F. B., Borini C. B. (2008). The mental foramen position in dentate and edentulous Brazilian's mandible. *International Journal of Morphology*.

[38] Anderson L. C., Kosinski T. F., Mentag P. J. (1991). A review of the intraosseous course of the nerves of the mandible. *Journal of Oral Implantology*.

[39] Cheung L. K., Leung Y. Y., Chow L. K., Wong M. C. M., Chan E. K. K., Fok Y. H. (2010). Incidence of neurosensory deficits and recovery after lower third molar surgery: a prospective clinical study of 4338 cases. *International Journal of Oral and Maxillofacial Surgery*.

[40] Valmaseda-Castellón E., Berini-Aytés L., Gay-Escoda C. (2002). Deflection of the mandibular canal near third molar roots and severe bleeding are associated with a significantly increased risk for inferior alveolar nerve damage original article. *Journal of Evidence Based Dental Practice*.

[41] Gargallo-Albiol J., Buenechea-Imaz R., Gay-Escoda C. (2000). Lingual nerve protection during surgical removal of lower third molars: A prospective randomised study. *International Journal of Oral and Maxillofacial Surgery*.

[42] Kipp D. P., Goldstein B. H., Weiss W. W. (1980). Dysesthesia after mandibular third molar surgery: a retrospective study and analysis of 1,377 surgical procedures. *Journal of the American Dental Association*.

[43] Carmichael F. A., McGowan D. A. (1992). Incidence of nerve damage following third molar removal: A West of Scotland Oral Surgery Research Group Study. *British Journal of Oral and Maxillofacial Surgery*.

[44] Falkine R. Z., Rossi A. C., Freire A. R. (2014). Relations between the mandibular canal and I, II and III angle classes in panoramic radiographs. *International Journal of Morphology*.

[45] Black C. G. (1997). Sensory impairment following lower third molar surgery: a prospective study in New Zealand.. *The New Zealand Dental Association Journal*.

[46] Bruce R. A., Frederickson G. C., Small G. S. (1980). Age of patients and morbidity associated with mandibular third molar surgery. *The Journal of the American Dental Association*.

[47] Ceballos F., González J., Hernández P., Deana N., Alves N. (2017). Frequency and position of the mental foramen in panoramic X-rays: Literature review. *International Journal of Morphology*.

[48] Fuentes R., Cantin M., Navarro P., Borie E., Beltran V., Bucchi C. (2014). Caracterización de Estructuras Anatómicas Mediante Radiografías Panorámicas: El Foramen Mental. *International Journal of Morphology*.

[49] Pyun J.-H., Lim Y.-J., Kim M.-J., Ahn S.-J., Kim J. (2013). Position of the mental foramen on panoramic radiographs and its relation to the horizontal course of the mandibular canal: A computed tomographic analysis. *Clinical Oral Implants Research*.

[50] Verma P., Bansal N., Khosa R. (2015). Correlation of radiographic mental foramen position and occulusion in three different indian populations. *The West Indian Medical Journal*.

[51] Chkoura A., El Wady W. (2013). Position of the mental foramen in a Moroccan population: A radiographic study. *Imaging Science in Dentistry*.

[52] Parnami P., Gupta D., Arora V., Bhalla S., Kumar A., Malik R. (2015). Assessment of the horizontal and vertical position of mental foramen in Indian population in terms of age and sex in dentate subjects by panoramic radiographs: A retrospective study with review of literature. *The Open Dentistry Journal *.

[53] Thakare S., Mhapuskar A., Hiremutt D., Giroh V. R., Kalyanpur K., Alpana K. R. (2016). Evaluation of the position of mental foramen for clinical and forensic significance in terms of gender in dentate subjects by digital panoramic radiographs conclusion: there was no difference in position of mental foramen in horizontal and vertical planes. *The Journal of Contemporary Dental Practice*.

